# Sustainable Earthquake Resilience with the Versatile Shape Memory Alloy (SMA)-Based Superelasticity-Assisted Slider

**DOI:** 10.3390/s22186876

**Published:** 2022-09-12

**Authors:** Peyman Narjabadifam, Mohammad Noori, Ertugrul Taciroglu, Jian Zhang, Behrokh Khoshnevis, Donatello Cardone, Dipanjan Basu, Tao Wang, Eltahry Elghandour, Ehsan Noroozinejad Farsangi, Reza Lotfi, Mahdi Chavoshi, Davood Sattarian, Orlando Fabio Stirnimann

**Affiliations:** 1 Department of Civil Engineering, Faculty of Engineering, University of Bonab, Bonab 5551395133, Iran; 2 Laboratory of Structural Earthquake Engineering (SEE-Lab), University of Bonab, Bonab 5551761167, Iran; 3 Department of Mechanical Engineering, California Polytechnic State University, San Luis Obispo, CA 93405, USA; 4 Department of Civil & Environmental Engineering, University of California, Los Angeles, CA 90095, USA; 5 Department of Industrial and Systems Engineering, University of Southern California, Los Angeles, CA 90089, USA; 6 Contour Crafting Corporation, El Segundo, CA 90245, USA; 7 School of Engineering, University of Basilicata, 85100 Potenza, Basilicata, Italy; 8 Department of Civil and Environmental Engineering, University of Waterloo, Waterloo, ON N2L 3G1, Canada; 9 Key Laboratory of Earthquake Engineering and Engineering Vibration, Institute of Engineering Mechanics, China Earthquake Administration, Sanhe 065201, China; 10 Department of Civil Engineering, The University of British Columbia (UBC), Vancouver, BC V6T 1Z4, Canada; 11 International Institute for Urban Systems Engineering, Southeast University, Nanjing 211189, China; 12 Department of Research and Development for Anti-Seismic Testing and Certification, Mageba SA, Solistrasse 68, 8180 Bülach, Switzerland

**Keywords:** shape memory alloy, resilience, earthquake, sustainability, aseismic isolation, structure, nonstructural systems, hospital, seismic protection, disaster prevention, shaking table

## Abstract

Earthquakes threaten humanity globally in complex ways that mainly include various socioeconomic consequences of life and property losses. Resilience against seismic risks is of high importance in the modern world and needs to be sustainable. Sustainable earthquake resilience (SER) from the perspective of structural engineering means equipping the built environment with appropriate aseismic systems. Shape memory alloys (SMAs) are a class of advanced materials well suited for fulfilling the SER demand of the built environment. This article explores how this capability can be realized by the innovative SMA-based superelasticity-assisted slider (SSS), recently proposed for next-generation seismic protection of structures. The versatility of SSS is first discussed as a critical advantage for an effective SER. Alternative configurations and implementation styles of the system are presented, and other advantageous features of this high-tech isolation system (IS) are studied. Results of shaking table experiments, focused on investigating the expected usefulness of SSS for seismic protection in hospitals and conducted at the structural earthquake engineering laboratory of the University of Bonab, are then reported. SSS is compared with currently used ISs, and it is shown that SSS provides the required SER for the built environments and outperforms other ISs by benefitting from the pioneered utilization of SMAs in a novel approach.

## 1. Introduction

**Figure 1:**
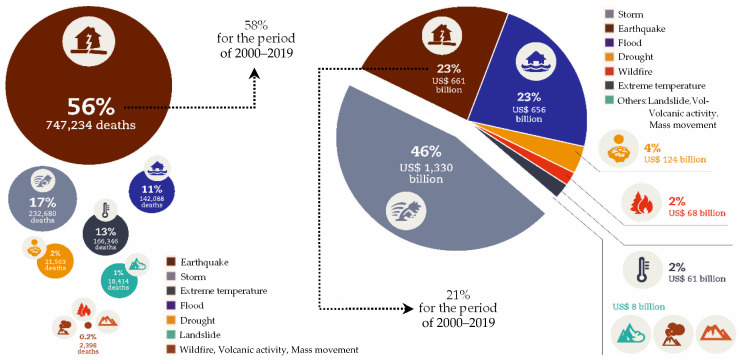
The continuing challenge of protection against earthquakes, indicated by comparing (**left**) the number of deaths and (**right**) the recorded economic losses due to different types of disasters occurred between 1998 and 2017 [4] together with the most recent updates for the earthquakes [3].

Seismic events are naturally certain to occur in earthquake-prone areas, distributed almost all over the world. Conquering nature to prevent the occurrence of earthquakes is technically unrealistic and seems daunting, at least in a near future. Realistic engineering approaches are required to achieve safety against earthquakes. Prediction of earthquakes can be considered as a realizable scenario but is somewhat complicated in practice and, in principle, does not provide a solution for the problem of safety against earthquakes. The rational solution that makes sense is improvement of the effectiveness of aseismic structural systems. While the achievements in developing seismic protective systems have been helpful in reducing the tragic consequences of earthquakes, recent researches indicate that earthquakes are still significant challenges to overcome [1,2,3,4]. Figure 1 is a brief representation of the continuing challenge of seismic protection, and demonstrates the need for further improvements to mitigate earthquake disasters.

A winning strategy toward overcoming the challenge should be the improvement of the anti-seismic systems that have shown satisfactory performance in prior earthquakes and can satisfy safety requirements mandated by seismic codes [5,6,7,8]. Statistics show that aseismic isolation (AI) is accepted as the most effective technique in protecting structures against earthquakes [9,10,11,12] but still requires an improvement to be appropriately effective in SER. Advanced materials can indeed be used to make the required improvement practically available. SMAs are a class of advanced materials suitable for this purpose [13,14,15,16,17]. Austenitic SMAs are preferred to martensitic ones, due to their favorable characteristics such as superelasticity with large strain plateaus, adequate energy dissipation capacity through flag-shaped hysteretic loops, and high fatigue and corrosion resistance [18,19,20,21,22].

**Figure 2:**
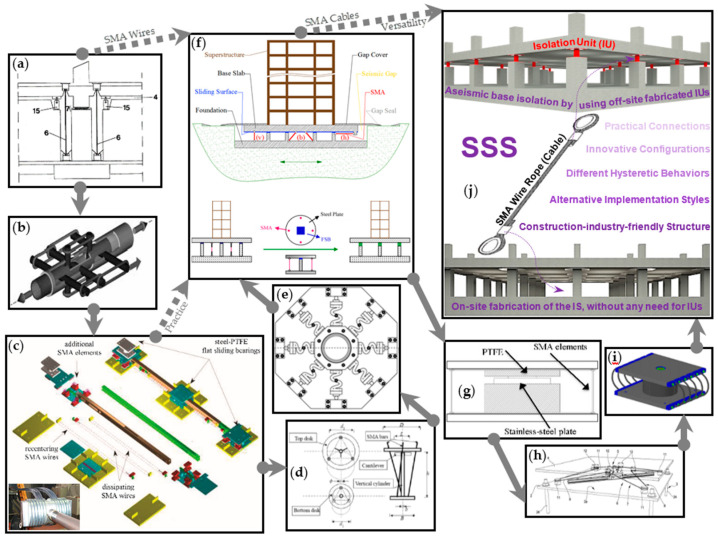
The history of SMA-based sliding ISs: (**a**) Logiadis et al. in 1997 [38]; (**b**) Dolce et al. in 2000 [41]; (**c**) Cardone et al. in 2003 [44]; (**d**) Casciati et al. in 2007 [45]; (**e**) Attanasi et al. in 2008 [46]; (**f**) Cardone et al. in 2009 [48]; (**g**) Ozbulut and Silwal 2014 [54]; (**h**) Colato and Castellano in 2015 [55]; (**i**) Zheng and Dong in 2017 [59]; (**j**) Narjabadifam et al. in 2020 [69].

SMA-based AI can be realized in many ways and different ISs can be used. Among the most promising ISs, rubber bearings have widely been implemented in practice [23,24,25,26,27], and sliding isolators have attracted a high attention due to their practical advantages, including: the further elongation of the natural period, the insensitivity to the frequency content of excitation, the lower transmission of accelerations into the superstructure, and the more robust stability due to the separate functions of carrying weight and providing isolation [28,29,30,31]. Various modifications of elastomeric and sliding ISs are available and aim at improving the effectiveness of AI. The effectiveness that can appropriately help toward a widespread SER is still lacking by existing ISs. This effectiveness can indeed be provided by flat sliding bearings (FSBs) which are the simplest sliders but possess higher degrees of the advantages of sliding isolators [32]. Principally, an FSB requires a proper restoring mechanism. Restoring mechanisms can typically be provided by combining the FSBs with laminated rubber bearings (LRBs). The friction pendulum system (FPS) and its variants are the other engineering solutions provided up to date. FPS has become a well-known practical IS due to its technical advantages. There are, however, some limitations with FPS and its variants [33,34]. SMA-based recentering is a relatively modern alternative solution for the restoring problem of sliding ISs [35,36,37,38,39,40,41]. Various SMA-based sliding ISs and SMA-based devices that can be used in sliding ISs or provide ideas for this purpose have been proposed by different researchers [42,43,44,45,46,47,48,49,50,51,52,53,54,55,56,57,58,59,60,61,62,63,64,65,66,67,68,69,70,71]. Widespread practical applications of SMAs in AI technology to improve its proven positive impact on SER, however, have not been possible because of some shortcomings of the existing systems. SSS has recently been proposed [72,73,74] to overcome some of these shortcomings due to its specifically pioneered structure, its construction-industry-friendly characteristics, and its other advantageous features such as the versatility provided by its alternative novel configurations. Figure 2 summarizes the aforementioned review, giving the attention only to the SMA-based sliding ISs, as SSS falls in this category and differs from other similar ISs that include SMA-based rubber bearings [75,76,77,78,79] and the ISs with steel cables [79,80,81,82,83].

The solid arrows connecting Figure 2a–j demonstrate the progress made by SSS and its significance. The IS patented by Logiadis et al. [42], represented in Figure 2a, basically materialized the proposal of SMA-based AI (Graesser and Cozzarelli in 1991 [84]) by combining FSBs (or LRBs) with auxiliary elements made either of iron or any alloy. A prototype of an SMA-based insulator (Figure 2b) was presented by Dolce et al. [45], Cardone et al. [48] proposed a mechanical system for SMA-based sliding isolation (Figure 2c), and the other related proposals (summarized in Figure 2d–i) were consequently formed by different invaluable ideas. SSS is an innovative IS that benefits from a pioneered structure to add versatility to its parent systems (Figure 2a,c,f), as noted on the arrows distinguished with the dashed lines in Figure 2. This article explores this versatility (in Section 2) and reports the results of shaking table studies (in Section 3) to reveal the capabilities of SSS for providing the built environment with SER. 

## 2. The Versatility of SSS

**Figure 3:**
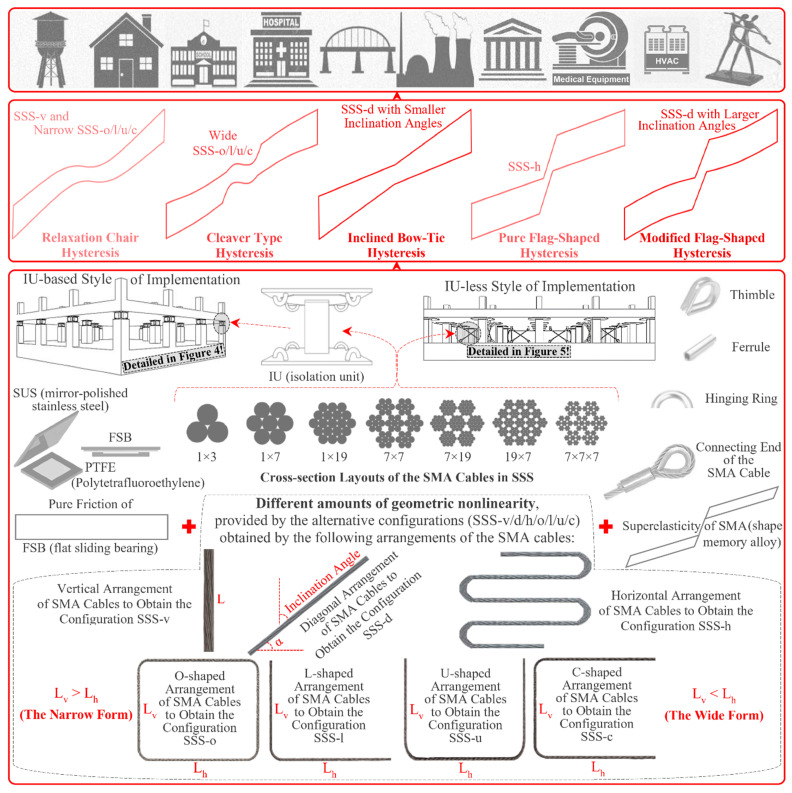
The versatility of SSS, at a glance.

Figure 3 provides an insight into the versatility of SSS, which makes it possible to practically benefit from the advantages of both SMAs and FSBs in SER through effective AI of a broad range of structures.

It is shown that the innovative utilization of SMA wire ropes (or cables) [85,86,87,88,89,90,91,92,93] as auxiliary isolating elements in an alternative novel and conventional configurations creates different hysteretic behaviors (relaxation chair, cleaver type, inclined bow-tie, modified flag shape, and pure flag-shaped hysteresis). These useful behaviors together with the pioneered practical structure of SSS are the main reasons for its versatility. As can be seen, different cross-section layouts of the SMA cables can be used in all the configurations (SSS-v/d/h/o/l/u/c, obtained by vertical/diagonal/horizontal/O-shaped/L-shaped/U-shaped/C-shaped arrangements of the SMA cables, respectively), and the cables can easily be replaced. Friction phenomenon can also be controlled through lubrication. All these, and the possibility for using different SMA and sliding materials, are the additional reasons for the versatility of SSS. The alternative isolation unit (IU)-based industrial and IU-less traditional styles of implementation, moreover, add to the versatility of SSS.

**Figure 4:**
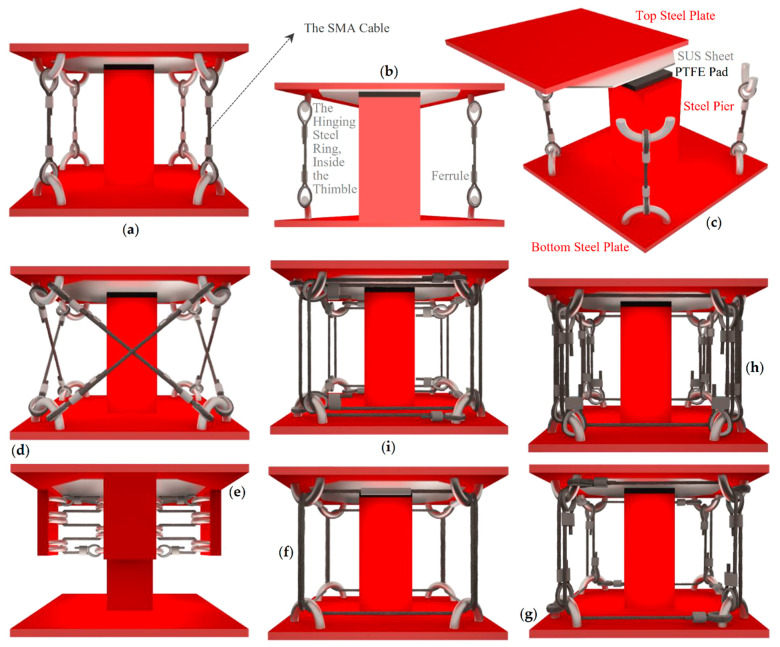
IUs of SSS: (**a**) the IU of SSS-v; (**b**) a cross section passing through a diagonal plane of the IU of SSS-v; (**c**) a disassembled view of the IU of SSS-v; (**d**) the IU of SSS-d; (**e**) the IU of SSS-h; (**f**) the IU of SSS-o; (**g**) the IU of SSS-l; (**h**) the IU of SSS-u; (**i**) the IU of SSS-c.

**Figure 5:**
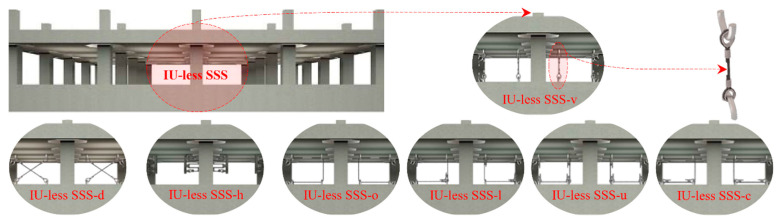
SSS in the IU-less traditional style of implementation.

The IU-based industrial and IU-less traditional styles of implementation are further detailed in Figure 4 and Figure 5.

In Figure 4, the IUs of SSS are illustrated for all its alternative configurations (SSS-v, SSS-d, SSS-h, SSS-o, SSS-l, SSS-u, and SSS-c). Additional details about the structure of the IUs are shown in Figure 4 (Figure 4b,c) by providing the disassembled view of the IU of SSS-v and a cross section pathing through the diagonal plane of the IU of this sample configuration, selected to represent the other ones. Presented in Figure 5 are the alternative configurations of SSS in its IU-less traditional style of implementation, assuming a multi-story building as an example from the broad range of structures that can be protected by SSS.

Based on the characteristics shown in Figure 3, Figure 4 and Figure 5, the key features of SSS that result in its versatility can be summarized as follows:

(*i*) practical combination of the advantageous sliding and superelasticity by utilizing the cables of austenitic SMAs through the application of the connecting devices that are mainly composed of thimbles and ferrules (and additionally allow for some useful operations such as prestressing and replacing the cables);
(*ii*) alternative novel and conventional configurations that provide this IS with the capability to adapt itself with various technical requirements of different projects, ranging from AI of a heavy large-scale structure such as a building to AI of a light-weight, small-scale structure such as an art object;
(*iii*) various hysteretic behaviors that can be obtained by the alternative configurations and their geometric variants to make the different performance objectives (e.g., higher isolation capability with the relaxation chair or cleaver type hysteresis and higher restoring capability with the pure flag-shaped hysteresis) achievable [69];
(*iv*) modularity that makes the system highly attractive in the practice of structural engineering and facilitates widespread AI;
(*v*) possibility for using different cross-section layouts of the cables depending on the levels of the forces (e.g., 1 × 3 for a light-weight, small-scale art object and 7 × 7 or maybe 7 × 7 × 7 for a heavy large-scale building);
(*vi*) possibility for working with any cross-section diameter of the SMA wires within the cables;
(*vii*) possibility for using different SMA materials or alloy compounds (e.g., the well-known NiTi-based alloys [14,36], the alternative cheaper and easy-to-fabricate Cu-based alloys [94,95,96,97,98], or the low-price Fe-based alloys [99,100,101,102,103,104] that are going to reduce the cost and increase the affordability of the SMA-based structural and earthquake engineering due to the improving metallurgical technologies);
(*viii*) possibility for using different sliding materials [105,106] and interfaces (e.g., the mostly used SUS-PTFE interface consisting of a mirror-polished stainless-steel plate on a polytetrafluoroethylene pad that can possibly include dimpled recesses for the purpose of lubrication [107], the SUS-PET interface that provides a relatively modern alternative for SUS-PTFE to be used in climatic regions [108] by replacing PTFE with self-lubricating thermoplastics blend of polyethyleneterephtalate (PET), the recently proposed [109] economic interfaces composed of different types of polyethylene, such as high-density polyethylene or ultrahigh molecular weight polyethylene sliding on galvanized steel);
(*ix*) alternative implementation styles (the IU-less traditional style of implementation, in addition to the IU-based industrialized style of implementation);
(*x*) capability to provide enhanced vertical isolation effect [74] through the utilization of some mechanisms or added elements of traditional or advanced materials (e.g., steel or SMA coil springs [110], super high damping rubber pads [111], viscous or steel dampers [112], telescoping piers [113], metallic or nonmetallic 3d/4d-printed metamaterial or periodic material pads [114], biomimetic architected elements [115], or combinations thereof).

Due to the versatility provided by the abovementioned advantageous features, SSS can offer unique opportunities in the field of structural engineering by providing a single platform for high-performance aseismic base isolation of a broad range of structures, as represented symbolically at the top of the Figure 3.

Below are the other technical advantages of SSS that, together with its versatility, help this innovative IS to improve SER in the built environment:

(*xi*) the passive control framework;
(*xii*) capability to monitor the health of the IS based on the self-diagnostic properties of the utilized SMA cables;
(*xiii*) capability to rehabilitate the IS by replacing its elements in cases of overloading;
(*xiv*) uplift resistance provided by the SMA component of the system;
(*xv*) fail-safe robustness provided by the sliding basis of the system;
(*xvi*) resistance to aging due to the superiority of SMAs also in this regard.

For the buildings, for example—as they are the largest group of the structures in the built environment that serve several societal needs, SSS can indeed provide effective earthquake protection through both earthquake-resistant design of new buildings and anti-seismic retrofit of existing ones. It can also be used for post-event rehabilitation and restoration, in addition to the aforesaid pre-event applications. The effect of SSS in seismic performance enhancement is further discussed in the next section based on the results of shaking table tests aimed at investigating seismic safety in hospitals that are a category of building structures with the highest importance in earthquake engineering. 

## 3. Shaking Table Studies

**Figure 6:**
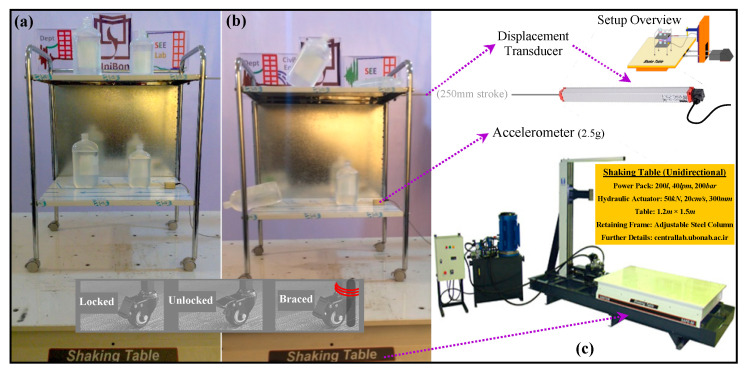
The hospital cart on the shaking table: (**a**) before and (**b**) during an experiment; (**c**) the technical details regarding the instrumentation and the mechanical system of the shaking table.

A hospital cart was tested on a shaking table to explore how SSS improves SER. The study of hospital cart was motivated by two technically important facts. First, the critical importance of hospital buildings relative to other types of buildings and structures in the built environment, as they need to be immediately occupiable and functional after an earthquake. Second, the high cost of hospital equipment and other sensitive contents in hospitals. The shaking table tests were conducted at the structural earthquake engineering laboratory of the University of Bonab. Figure 6 shows the cart on the shaking table, both before and during an experiment, and provides the required technical details regarding the instrumentation and the mechanical system of the shaking table. 

The cart was supported on four twin wheel casters with common brakes, and two large (1 litter) and small (0.5 litter) plastic bottles of sterile saline serums were carried on both of its top and bottom trays.

**Figure 7:**
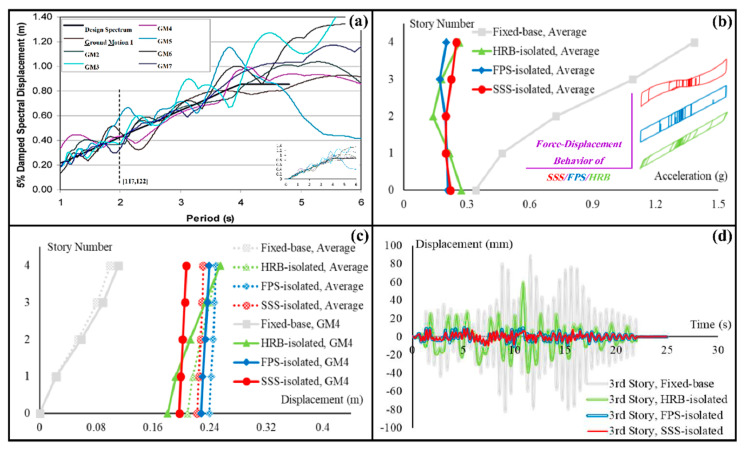
Production of the input for the shaking table experiments: (**a**) response spectra of the ground motion records used in this study, and the reference design spectrum; (**b**) average maximum story accelerations; (**c**) average maximum story displacements and maximum story displacements under the selected ground motion record; and (**d**) the input excitations produced for the experiments.

Regarding the specific technical requirements of the research, a cascading approach was followed and nonlinear time history analyses of a theoretical four-story reinforced concrete moment-resisting-frame hospital building with plan dimensions of 15 m by 15 m and 3.1 m height per story with masses equal to 225 tons resulting in the fundamental period of 0.5 s were performed in the well-known SAP2000 software [116] to produce the input for the shaking table experiments. One fixed-base and three base-isolated forms of the building subjected to a set of seven ground motion records, suggested by the Italian network of seismic engineering laboratories [117] for the studies of ISs, were analyzed. According to the technical requirements [118,119,120,121], the ground motion records were scaled to match, on average, the relevant design spectrum. The ISs investigated in the analyses were SSS, FPS, and high-damping laminated rubber bearing (HRB). Story acceleration and story displacement histories were obtained under all the ground motion records. One of the records was selected to produce the required input, and the displacement history of the third story of the building under this ground motion record was prepared for the experiments. Figure 7 summarizes the process discussed above. The preparation of the seismic action for the periods of interest in AI, i.e., >2 s [122] is represented in Figure 7a. The seismic performances are summarized in Figure 7b,c by reporting the profiles of maximum story accelerations and displacements on average over the seven ground motion records (GM1 to GM7). Figure 7c also breaks the average responses down to the share of GM4, which—because of better representing the average responses [123,124,125]—was selected for the shaking table experiments. The input excitations produced for the experiments, i.e., the displacement histories of the third story in all fixed-base and base-isolated forms of the hospital building subjected to GM4 are shown in Figure 7d.

Comparisons between the profiles of story displacements and accelerations in the SSS-isolated condition with those in the FPS and HRB-isolated conditions, summarized in Figure 7b,c, indicate the potential of SSS to improve SER by providing a high-quality alternative to the currently used ISs in protecting the primary structural systems. The competitive seismic performances are indeed additional advantages to the versatility discussed in the previous section. In addition to the fact that SSS can also be used for the AI of equipment and contents, earthquake protection of the secondary structural systems and the nonstructural secondary systems through the enhancement of the performances of the primary structural systems can typically be explored by applying the excitations illustrated in Figure 7d to the hospital cart shown in Figure 6a.

**Figure 8:**
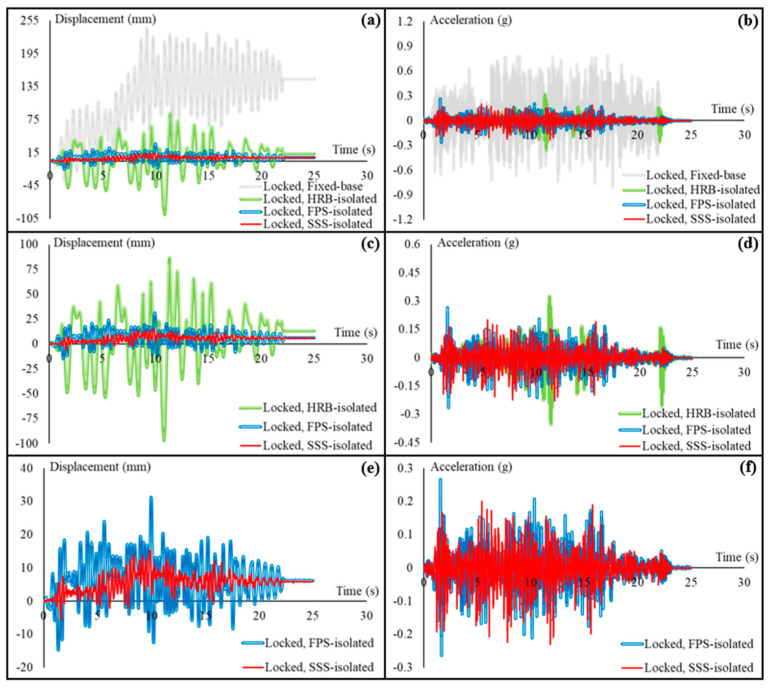
Seismic responses of the cart with locked wheels on the shaking table: (**a**) comparison of the displacement responses in the fixed-base and base-isolated hospital building; (**b**) comparison of the acceleration responses in the fixed-base and base-isolated building; (**c**) displacement response comparison between the isolated cases; (**d**) acceleration response comparison between the isolated cases; (**e**) SSS compared with FPS (as the other sliding IS), in terms of the recorded displacement response of the cart; and (**f**) SSS compared with FPS, in terms of the acceleration response.

**Figure 9:**
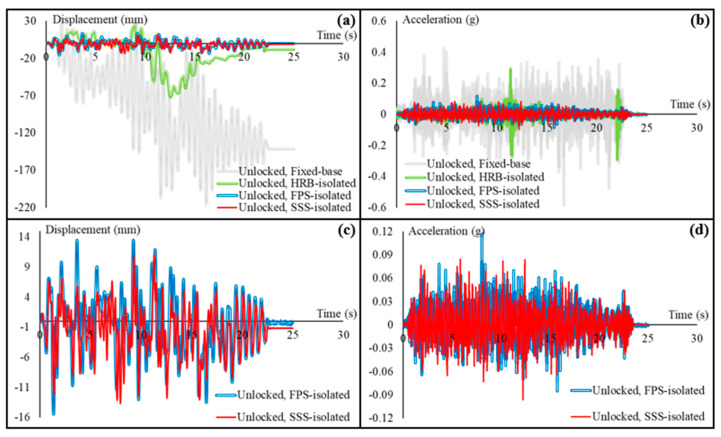
Seismic responses of the cart with unlocked wheels on the shaking table: (**a**) comparison of the displacement responses in the fixed-base and base-isolated hospital building; (**b**) comparison of the acceleration responses in the fixed-base and base-isolated building; (**c**) SSS compared with FPS, in terms of the recorded displacement response of the cart; and (**d**) SSS compared with FPS, in terms of the acceleration response.

**Figure 10:**
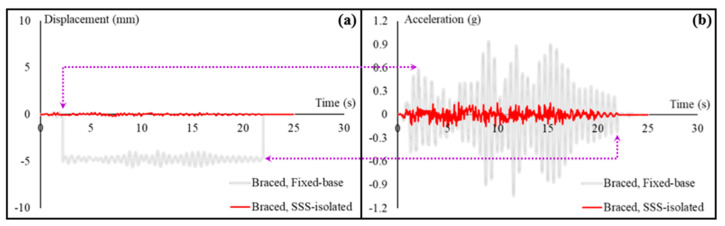
Seismic responses of the cart in the braced condition on the shaking table: (**a**) comparison of the displacement responses in the fixed-base and SSS-isolated hospital building; (**b**) comparison of the acceleration responses in the fixed-base and SSS-isolated building.

**Figure 11:**
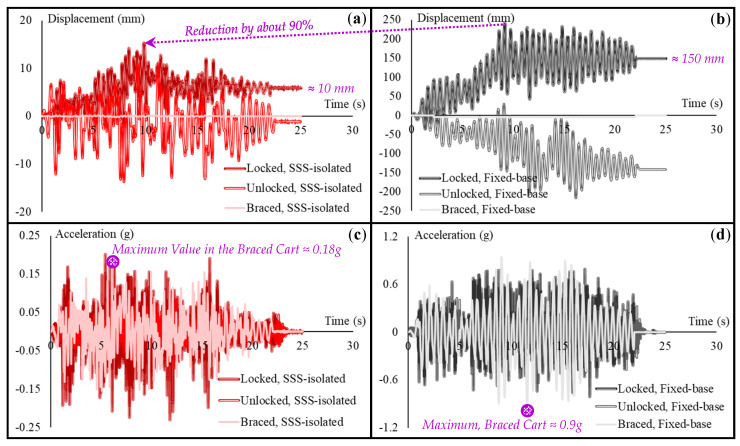
Comparative diagrams of the seismic responses of the cart in the locked, unlocked, and braced conditions: (**a**) displacement responses in the SSS-isolated case; (**b**) displacement responses in the fixed-base case; (**c**) acceleration responses in the SSS-isolated case; (**d**) acceleration responses in the SSS-isolated case.

**Figure 12:**
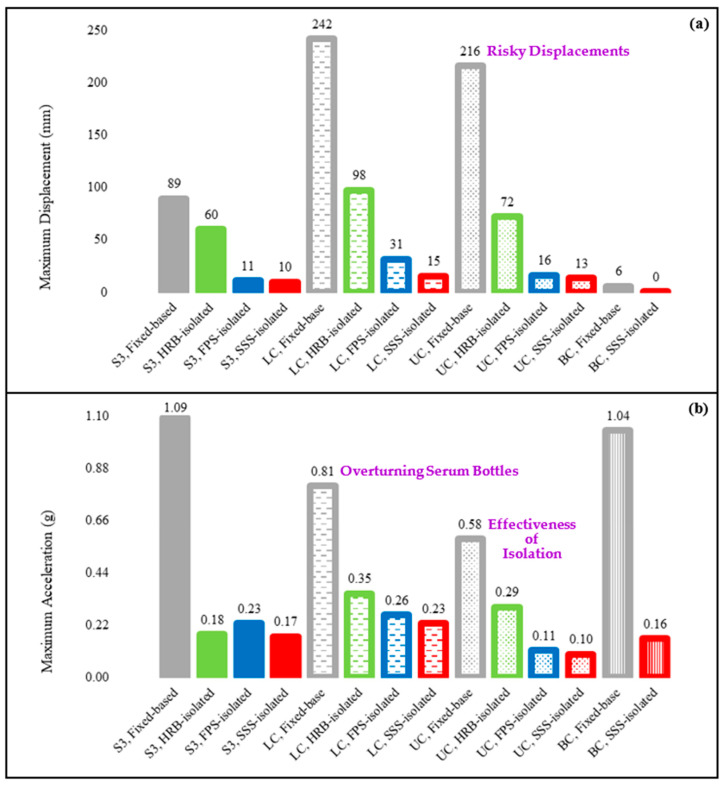
Maximum (**a**) displacements and (**b**) accelerations of the ground motion GM4 (used to generate the input excitations for the shaking table tests), the third story (S3) of the hospital building in all the fixed-base and base-isolated (HRB, FPS, and SSS-isolated) cases subjected to GM4, and the cart in all the locked (LC), unlocked (UC), and braced (BC) conditions in the fixed-base and base-isolated cases.

The cart has been tested in locked, unlocked, and braced, i.e., loosely fastened to two anchor bolts on the table, conditions subjected to the displacement response histories of the third story of the fixed-base and HRB/FPS/SSS-isolated building. Results obtained from the tests have been summarized in Figure 8, Figure 9, Figure 10, Figure 11 and Figure 12. Figure 8 shows the displacement and acceleration responses of the cart with locked wheels, comparing them in the fixed-base and base-isolated buildings. Figure 8a compares the displacement responses in all the isolated cases and the fixed-base case. Figure 8b makes the same comparisons possible for the accelerations. In order to better understand the differences between the isolated performances, the fixed-base case is removed in Figure 8c,d. Figure 8e,f, and, more specifically, SSS is compared to the other isolation-based IS (FPS). 

Based on the comparisons provided in Figure 8a,b, aseismic base isolation of the primary structural system of the hospital building effectively reduces both the displacement and acceleration responses of the cart that represents nonstructural systems. Figure 8a clearly shows that the displacement of the cart with locked wheels in the fixed-base building is much higher than those in the isolated building, and the cart in the fixed-base building remains around 150 mm displaced once earthquake has ended, while—independent of the type of the IS—the cart stays almost at the same place in the base-isolated building. It should be noted that the reported displacements of the cart are relative displacements, calculated from the recorded absolute displacements. Similarly, the effectiveness of aseismic base isolation of the primary structural system is evident in Figure 8b, showing how the accelerations are attenuated. As expected, due to the technical advantages mentioned in the introduction section, by comparing the performances only between the ISs (Figure 8c,d) it is clear that the sliding ISs (SSS and FPS) perform better. Figure 8e,f are additionally provided to more precisely investigate the competitiveness of SSS, which is clear when the maximum displacement equal to 31 mm observed in the FPS-isolated building is compared with 15 mm in the SSS-isolated building. As for the accelerations, the maximum acceleration of the cart in the SSS-isolated building is 0.23 g, while it is approximately 15% higher in the FPS-isolated building.

Figure 9a–d summarizes the experimental results obtained by testing the cart in the unlocked condition. Seismic responses of the cart in all fixed-base and base-isolated cases are compared in Figure 9a (displacements) and 9b (accelerations), and more clear comparisons between the two sliding systems are provided in Figure 9c,d.

Both maximum displacements and accelerations in the unlocked condition of the cart are lower than those in the locked condition, for all fixed-base and base-isolated cases. Residual displacement of the cart after the earthquake in the unlocked condition subjected to the seismic motions in the fixed-base building, however, is almost same as that in the locked condition. Aseismic base isolation of the primary structural system is effective in terms of seismic safety of the cart, also in the unlocked condition. The sliding ISs are again more effective, and SSS provides better performances.

For the braced condition of the cart, both the displacement and acceleration responses are compared in Figure 10 between the fixed-base case and the SSS-isolated case that is the most effective IS according to the observations obtained from the time history analyses and the shaking table tests. Figure 10a shows that the cart experiences a partial release of around 5 mm because of the intensive excitation in the fixed-base case, while almost zero displacements are experienced by the cart in the SSS-isolated case. These observations are verified by the acceleration responses in Figure 10b. The double-arrow orange lines, for example, explain why the 5-mm release is experienced.

In order to compare all the locked, unlocked, and braced conditions together, Figure 11 presents comparative diagrams of the displacement and acceleration responses of all these conditions in the SSS-isolated and fixed-based cases.

As seen in Figure 11a,b, SSS-based AI of the primary structural system of the building reduces the maximum displacements of the cart with locked and unlocked wheels by about 90% with respect to those in the fixed-base building. The effectiveness of SSS, and the other ISs compared with SSS in Figure 7, Figure 8 and Figure 9, however, becomes more evident when the residual displacement of the cart at the end of the seismic motions is concerned as another critical criterion. A residual displacement of around 150 mm in both locked and unlocked conditions in the fixed-base case reduces to 10 mm in the locked condition in the SSS-isolated case which prevents the residual displacement in the unlocked condition. Even if the residual displacement of the cart is almost zero in the braced condition in both fixed-base and SSS-isolated cases, comparison of the acceleration responses in Figure 11c,d shows that accelerations experienced in the fixed-base case are about 5 times of those in the SSS-isolated case. SSS reduces the accelerations also in the locked and unlocked conditions, with the highest reductions in the unlocked condition. Based on the comparisons in Figure 11, it can be claimed that aseismic base isolation of the primary structural system by using SSS prevents large displacements and high accelerations experienced by the cart (and other nonstructural systems typically represented by the cart) in an earthquake. However, both displacements and accelerations experienced in the fixed-base hospital building are risky in terms of seismic safety. Even if the displacements are prevented in the braced condition of the cart in the fixed-base case, the high accelerations (almost same as those experienced by the floor) are dangerous. This was indeed observed in the shaking table tests, when both small and large bottles of the saline serums overturned in both trays of the cart. A similar situation was observed in the locked condition (shown in Figure 6b) due to the high accelerations. Displacements are also large in this condition and the cart remains around 150 mm displaced at the end of the earthquake. These large displacements can themselves be dangerous, as the moving cart can crash into other sensitive equipment and hurt patients or the hospital personnel. The accelerations in the unlocked condition of the cart in the fixed-base case are relatively lower but the large displacements are again risky.

The discussions above indicate that SSS provides an effective protection against earthquakes in all locked, unlocked, and braced conditions, while the seismic safety can be at risk in the fixed-base case. Based on the comparisons, similar effectiveness can be obtained by other ISs. These effects can be better understood by comparing the maximum responses, which are reported in Figure 12. According to Figure 12, it is clear that AI of the primary structural system prevents damaging accelerations and risky displacements to be experienced also by nonstructural systems represented by the hospital cart in this study. It is also seen that SSS has the highest effectiveness among the other ISs. The other significant observation is the effectiveness of the AI of equipment and contents, which is demonstrated by the lower accelerations experienced in the unlocked cart, noting the fact that large displacements can effectively be controlled and restored using an IS such as SSS. The modularity of SSS and its alternative configurations, which were discussed in Section 2, make these kinds of applications easily possible to further improve SER in the built environment. The price of SMA is expected to not be a problem soon because of significant continuous decrease in price due to improving metallurgical technologies and the possibility of applying low-price, high-performance, Iron-based or other superelastic alloys. In this regard, the interest in SMA-based AI is continuously increasing and different systems (e.g., [126,127]) are being proposed.)

## 4. Conclusions

This paper presented the results of an experimental investigation performed to verify the effectiveness of advanced AI (aseismic isolation) technology and SMA (shape memory alloy)-based anti-seismic structural design in SER (sustainable earthquake resilience). The versatile SSS (SMA-based superelasticity-assisted slider), recently proposed to improve the positive impact of AI on SER, represented the advanced AI technology and SMA-based design. A concise problem statement along with a comprehensive literature review on both AI and SER was included in the introduction section of the paper to provide the necessary background for the discussions. Subsequently, an analytical study was carried out to explore how SSS provides a versatile alternative to currently used ISs (isolation systems). The versatility of this innovative and high-tech IS was carefully assessed based on its mechanical characteristics. Technical drawings of its alternative configurations and implementation styles were presented, and its valuable features were described. It was shown how the pioneered structure of SSS makes the practical implementation possible in the IU (isolation unit)-less style in addition to the IU-based style. It was also discussed how the alternative configurations of SSS make various performances achievable by the alternative hysteretic behaviors, how the pioneered structure of this IS provides multiple design options in different projects, how the modularity of SSS facilitates rehabilitation in the case of possible overloading, and how the application of the SMA cables materializes the technically advantageous self-diagnosis that can be utilized for the purpose of health monitoring. Finally, as the central part of the paper, the shaking table experiments and their results were discussed. The basic information about the setup, testing facility, and measurement devices was provided, and the nonlinear time history analyses executed to produce the input excitations for the shaking table tests were outlined. HRB (High damping laminated rubber bearing) and FPS (friction pendulum system) were compared with SSS. It was shown how the seismic performances of the primary structural system in a hospital building affect the responses of nonstructural systems that were represented by the study of a cart in the locked, unlocked, and braced conditions. Separate diagrams were presented for the displacement and acceleration responses of the cart in all three afore-mentioned conditions subjected to the displacement time histories of the third story of the hospital in all fixed-base and HRB, FPS, and SSS -isolated cases. Comparative diagrams were additionally presented to enrich the discussions by providing further information, for example, about the maximum seismic responses. The first significant observation was the effective reduction of both displacements and accelerations in all base-isolated cases. It was shown how the reduction in displacements is important in seismic safety, and how the reduced accelerations prevented the saline serum bottles from being overturned in both trays of the cart on the shaking table. The second significant observation reported herein was the verification of the higher SER-related effectiveness of the sliding-based ISs, which was expected due to the accepted technical advantages of these systems. The final significant observation was the competitiveness of SSS because of its higher performances in addition to its versatility provided by its pioneered structure.

## 5. Patents

Shape memory alloy (SMA)-based superelasticity-assisted slider (SSS) is a patented structural system (US Patent 11,313,145 [74]) which provides an innovative engineering solution to practically benefit from the valuable unique advantages of both friction and superelasticity in aseismic isolation to improve seismic resilience, and its sustainability in the built environment in the whole world.
